# Effect of Iron Content on Corrosion Properties of Pure Titanium as Grain Refiner

**DOI:** 10.3390/ma14237193

**Published:** 2021-11-25

**Authors:** Bosung Seo, Hyeon-Tae Im, Ki-Beom Park, Kwangsuk Park, Hyung-Ki Park

**Affiliations:** Gangwon Regional Division, Korea Institute of Industrial Technology, Gangneung-Si 25440, Gangwon-Do, Korea; bs3863@kitech.re.kr (B.S.); iht89@kitech.re.kr (H.-T.I.); hope92430@kitech.re.kr (K.-B.P.)

**Keywords:** pure titanium, titanium cathode, iron, grain refiner, corrosion

## Abstract

Microstructures and corrosion properties of pure titanium were characterized when iron was used as a grain refiner. The added Fe element acted as a strong grain refiner for pure titanium by forming β Ti phase at grain boundaries, and 0.15 wt% Fe was revealed to be a sufficient amount to make the grain size of pure titanium below 20 μm, which was the requirement for the desired titanium cathode. However, corrosion resistance was decreased with the Fe amount added. From the open circuit potential (OCP) results, it was obvious that the TiO_2_ stability against the reducing acid environment was deteriorated with the Fe amount, which seemed to be the main reason for the decreased corrosion resistance. Electrochemical impedance spectroscopy (EIS) results showed that both the decrease in the compact oxide film’s resistance (R_b_) and the appearance of the outer porous film occurred as a result of the dissolution of the TiO_2_ layer, whose phenomena became more apparent as more Fe was added.

## 1. Introduction

Titanium and titanium alloys have been used in corrosive environments, such as seawater and hazardous gases, due to their excellent corrosion resistance [1,2,3,4]. However, pure titanium would suffer from corrosion when exposed to a high-temperature chloride solution and non-oxidizing acid solutions with low pH [5,6,7,8]. Titanium cathode has commonly been utilized in producing copper foil for lithium ion batteries with a form of drum, where highly concentrated sulfuric acid is used as an electrolyte, indicating that corrosion would be an issue. It was reported that a grain size as small as ASTM grain size number 7 (~32 μm) is recommended for a titanium drum to suppress the formation of titanium hydride on the titanium drum [9]. The increased grain boundary as a result of small grain size could provide enough of a pathway to enable evolved hydrogen on a titanium drum during electrodeposition of Cu to diffuse into a titanium matrix.

Iron exists in pure titanium as an impurity, and its content is usually limited to 0.3 wt%, as it could affect microstructure in terms of β phase stabilization and/or Ti_x_Fe intermetallic formation [10]. These microstructural changes induced by the addition of Fe would be of use in refining grain size. Yan et al. reported that Fe content at the level of 1280 ppm enabled the formation of a grain boundary (GB) β Ti phase. In addition, a trace amount of P (<50 ppm) induced Ti-P phase at the grain boundary, which played the role of a grain growth inhibitor [11]. Similarly, grain boundary segregation was revealed to be an effective grain refinement method, where local enrichment of solute atoms at grain boundary occurs to reduce free energy, and finally, inhibit grain growth during heat treatments such as recrystallization [12]. Therefore, secondary phase formation at grain boundary such as β Ti phase would stabilize grain boundary, and grain refinement could be achieved by controlling Fe content.

Not only microstructure, but also corrosion properties of titanium and titanium alloys were altered with Fe content. Schutz et al. showed that iron levels up to 0.3 wt% have no detectable impact on corrosion resistance of pure titanium in hot neutral, oxidizing, or oxidizing chloride acid environments [13]. He et al. reported that the crevice corrosion properties of Grade-2 titanium were significantly influenced by Fe content. Medium Fe content, as much as 0.078 wt%, deteriorated the corrosion performance of Grade-2 titanium by forming Fe-stabilized β Ti phase, whereas high Fe content of 0.12 wt% improved crevice corrosion resistance as a result of the formation of Ti_x_Fe intermetallic particles, which act as a catalyst for proton reduction. Increase in grain boundary caused by grain refinement could alter corrosion properties [14]. The previous studies demonstrated that grain size exerts a positive or negative impact on corrosion resistance depending on the materials’ passivation ability. In the case that passivation of titanium and titanium alloys is stable, the corrosion resistance of them would increase with grain refinement, because the small grain is prone to form a uniform passivation film [15,16,17]. However, exposed to the high concentrated sulfuric acid which provides the reduction environment, the passivation ability of titanium and titanium alloys would become weak, resulting in the decrease in corrosion resistance with grain refinement.

In this study, microstructures and corrosion properties of pure titanium (Grade 1) were investigated by varying Fe content as a grain refiner. As titanium cathode electrode for producing copper foil is operated under a highly concentrated sulfuric acid, understanding the variations of microstructures and resultant corrosion properties with Fe content is necessary for good performance of a titanium cathode, especially for a reduced acid environment.

## 2. Materials and Methods

The ingots of Ti_x_Fe (x = 0.03, 0.15, 0.3, 0.5, all in wt%) alloys were fabricated by vacuum arc melting, where remelting was carried out five times to obtain a homogenized chemical composition. Table 1 shows the chemical composition of each of the Ti_x_Fe alloys. Homogenization heat treatment was conducted at 1000 °C for 10 h in a high-vacuum atmosphere (10^−5^ torr), and then, hot rolling (80% reduction in thickness) was applied at 800 °C, leading to 1.6 mm thick plates. Finally, the plates were annealed at 750 °C for 60 min, allowing for sufficient recrystallization.

Thermo-Calc Software (Thermo-Calc Software, Stockholm, Sweden) was used to calculate equilibrium phases for the given TiFe compositions, and the actual phases were confirmed by X-ray diffraction (XRD, PANalytical, Almelo, Holland) with Cu K_α_. Grain was analyzed through electron backscatter diffraction (EBSD), where Orientation Imaging Microscopy (OIM) software (EDAX, Mahwah, NJ, USA) was used to obtain accurate grain size.

All electrochemical tests (Autolab PGSTAT302N, Metrohm Autolab, Utrecht, The Netherlands) were conducted with a three-electrodes configuration cell, where the exposed area of the working electrode was 100 mm^2^, and Pt plate and Ag/AgCl electrodes were used as counter and reference electrodes, respectively. Prior to the electrochemical tests, nitrogen gas was purged into the 30 wt% H_2_SO_4_ solution for 20 min. to remove dissolved oxygen. Corrosion properties were characterized by open circuit potential (OCP), potentiodynamic polarization, and electrochemical impedance spectroscopy (EIS) methods. Polarization tests were conducted to obtain Tafel plots, with a scan rate of 0.05 mV/s in the range from −1.5 V to 1.5 V. Electrochemical impedance spectroscopy (EIS) tests were performed at the open circuit potential with the AC amplitude of 10 mV over the frequency range from 10,000 to 0.05 Hz.

## 3. Results

### 3.1. Microstructure

**Figure 1:**
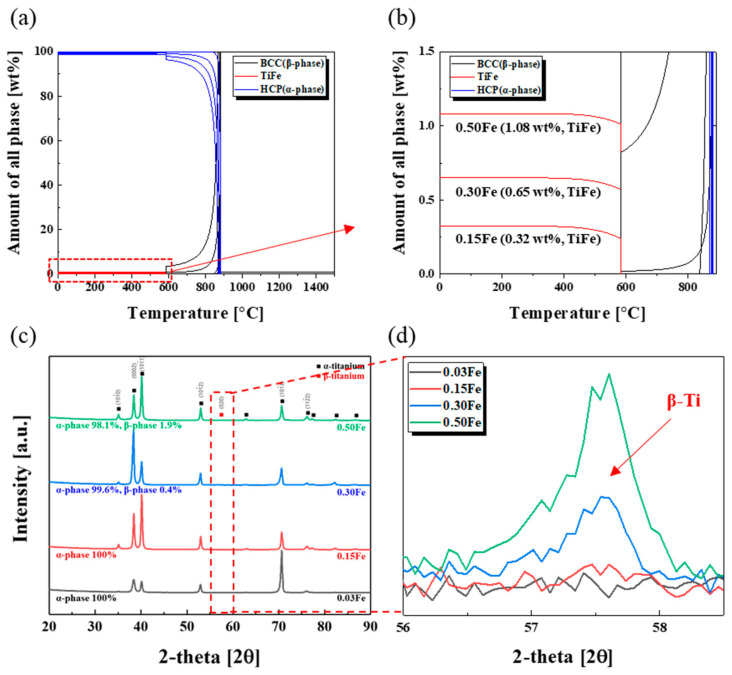
(**a**) Calculated phase diagram of TiFe alloys, (**b**) enlarged part showing variations of amount of the TiFe phase with Fe content, (**c**) XRD diffraction patterns of TiFe alloys where α and β Ti phases coexisted, and (**d**) enlarged part indicating the growth of the peak corresponding to β Ti phase with Fe content.

Figure 1a,b shows equilibrium phases and variations of their relative quantities with a function of Fe content calculated by Thermo-Calc software, where the mixture of α Ti and TiFe phases was revealed to be thermodynamically stable for pure Ti containing up to 0.5 wt% Fe. As a β stabilizing element, a β Ti phase became dominant at high temperature over 590 °C with the Fe content, and only a TiFe phase formed below 590 °C, indicating that this amount of Fe was not enough to form and/or stabilize β Ti phase at room temperature. However, it was reported that Fe content as low as 0.1 wt% could stabilize β Ti phase for pure titanium, where the Fe content in the formed β Ti phase was known to be about 6.5 wt% [13]. The other study also showed that the Fe composition in the β Ti phase ranged from 13.9 to 15.7 wt% depending on cooling rates, even though the Fe content was just 0.14 wt%, suggesting that local enrichment of the Fe element was needed for the formation of β Ti phase [18]. The XRD analysis shown in Figure 1c,d indicates that the β Ti phase started to form when the Fe content reached around 0.3 wt%, and its amount reached about 1.9 wt% when the Fe content increased up to 0.5 wt% (actually, the β Ti phase started to be formed around 0.2 wt% Fe, as shown in Supplementary Materials Figures S1 and S2). Even though TiFe phase was known to be stable thermodynamically, no peak corresponding to TiFe phase appeared in the XRD results. It was reported that isolated Ti_x_Fe particles were detected by SIMS imaging for the Ti-0.078 wt% Fe system, but they might be too tiny to be detected by the XRD method [14].

**Figure 2:**
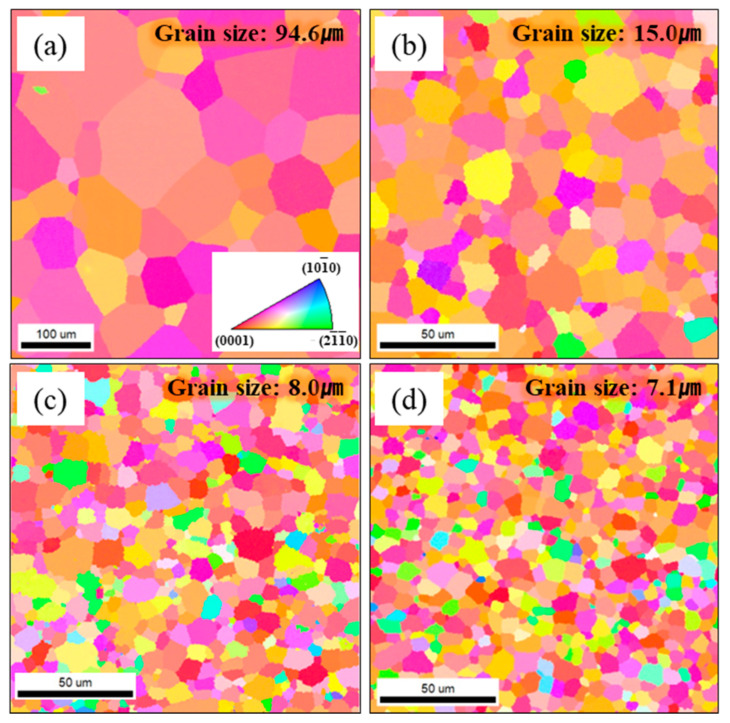
Orientation image maps showing grain size of (**a**) Ti-0.03Fe, (**b**) Ti-0.15Fe, (**c**) Ti-0.3Fe, and (**d**) Ti-0.5Fe.

Figure 2 displays variations of the grain size with Fe content. Although the final recrystallization condition was the same for all samples, the grains became refined with the Fe amount added, meaning that Fe could be available as grain refiner. Secondary phases such as TiFe and β Ti phases formed due to the presence of the Fe element, which would restrict grain growth during recrystallization [19]. As a result, the requirement of the grain size for a titanium cathode electrode could easily be achieved by only adjusting the Fe content between 0.15 and 0.3 wt%, values that fall in the high limit of the Fe content for a pure titanium such as Gr1, Gr2, and Gr3.

### 3.2. Corrosion Properties

**Figure 3:**
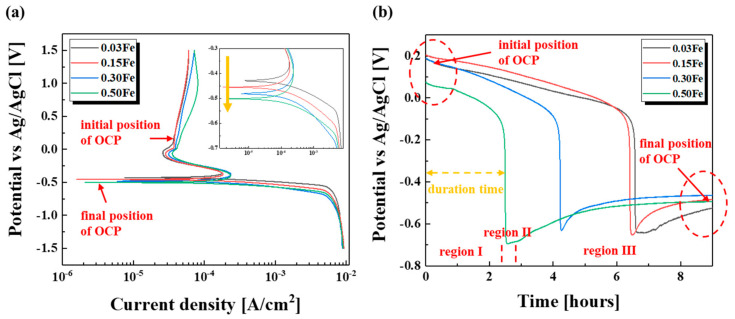
(**a**) Polarization curves and (**b**) variations of OCP with time for TiFe alloys. The inlet in Figure 3a is the enlarged part of the corrosion potential region.

As corrosion properties are influenced by microstructures as well as chemical compositions, the addition of Fe could alter the corrosion properties of the pure titanium in terms of grain refinement and Fe content. Figure 3a showed the polarization curves displaying an obvious passivation region, indicating that the TiFe alloys were well passivated even under the highly concentrated sulfuric acid environment. The curves of all samples looked almost the same in terms of corrosion potential, corrosion current density, and passivation region. However, when looking closely at the corrosion potential region (nose), the corrosion potential slightly moved toward the base direction with added Fe, which would be ascribed to the formation of the β Ti phase. Figure 3b showed the variations of OCP with the immersion time. All samples displayed similar behavior with time, where three distinct regions existed: gradual decrease, abrupt decrease, and stabilization in OCP. It is known that the abrupt decrease in OCP denoting passivation breakdown was ascribed to chemical dissolution of the TiO_2_ layer [20,21,22]. The Fe amount did not give an impact on the potential positions in each region of OCP, except for the duration time in the first region. The potential in the first region was located in the passivation region and the potential in the third one positioned around the corrosion potential based on the polarization results. As the first region lies in the passivation region, the duration time could imply passivation stability against the acid electrolyte, meaning that shorter duration time denoted poor passivation stability. Therefore, the higher Fe content, which had the shorter duration time, deteriorated the passivation stability. The gradual destruction of the TiO_2_ compact layer with the immersion time would cause loss in protective ability of the TiO_2_ film, resulting in the abrupt drop in OCP. After the decrease of OCP, the potential became stabilized around the value close to the corrosion potential, indicating that the TiFe alloys kept being placed under corrosion.

**Figure 4:**
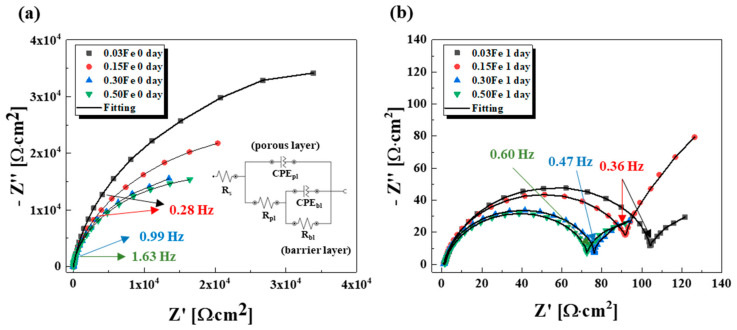
Nyquist plots measured at (**a**) 0 day and (**b**) 1 day for TiFe alloys. The inlet in Figure 4a is the equivalent circuit used to fit the data.

Electrochemical Impedance Spectroscopy (EIS) is a powerful tool in investigating corrosion properties of metals and their oxide films, where an equivalent circuit is used to interpret EIS spectra. Figure 4 displays the evolution of Nyquist plots with the immersion time, and the fitted data with the equivalent circuit related to two-layered structure (porous layer + barrier layer) are shown in Table 2. As shown in Figure 4a, the initial Nyquist plots presented single broad capacitive loops for all TiFe alloys due to the higher resistance of the barrier layer compared to that of the porous layer. The resistance of the barrier layer slightly decreased with increasing Fe content, indicating that adding more Fe into Ti deteriorated the corrosion resistance of the TiFe alloys. This trend in the resistance remained after 1 day immersion. With the immersion time, not only shrinkage of the loop diameter, but change of the loop shape occurred, from one single loop to two semicircles.

## 4. Discussion

As titanium and titanium alloys are well passivated, it is expected that grain refinement itself would exert a positive impact on corrosion resistance. However, the Fe element, which was added as a grain refiner, also lead to formation of a β Ti phase, which would alter the corrosion resistance. It was known that the less noble β Ti phase which usually forms at the grain boundary contributed to intergranular corrosion attack, so that the increased grain boundary would hurt the corrosion resistance in the case of the presence of the β Ti phase at the grain boundary. Even though the polarization results did not show significant differences in corrosion properties in terms of corrosion potential and corrosion current density, the OCP results provided obvious evidence of the deteriorated effect of the Fe element on corrosion properties. As shown in Schutz’s work where solid solution iron had no effect on the formation and/or stability of titanium oxide film, the passivation ability itself seemed not to be hurt by the addition of the Fe element from the initial position of OCP [23]. However, under the non-oxidizing acid condition such as the 30 wt% sulfuric acid electrolyte, recovery of a passive film would not be sufficient, leading to the gradual decrease in OCP with immersion time, as shown in Figure 3b. As denoted in the XRD results, more β Ti phase formed with adding of the Fe element, making the TiFe alloys susceptible to corrosion. As a result, the duration time of OCP denoting preservation of the passivation state became shorter with the Fe content. From the fact that the final OCP values of the Ti-Fe alloys were close to the corrosion potential, it can be inferred that no repassivation occurred due to the reducing acid condition. This depletion of repassivation ability in the reducing environment would weaken the positive impact of the refined grain on corrosion resistance. This means that the Fe addition as a grain refiner would deteriorate the corrosion resistance of pure Ti, because the negative effect of the Fe addition on corrosion performance, such as β Ti formation, was dominant.

**Figure 5:**
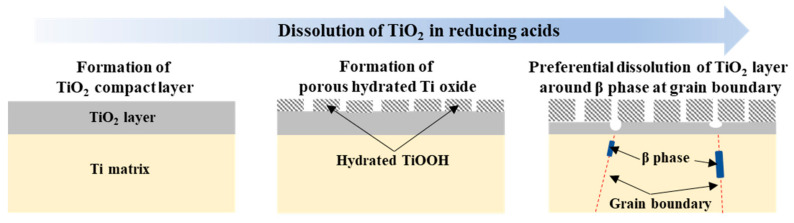
Schematic illustrating evolution of TiO_2_ layer as a result of dissolution of TiO_2_ in a reducing acid condition.

As the Nyquist spectra were divided into two semi-circles with immersion time, the equivalent circuit used in this study was constructed based on a two-layer model of an oxide film. R_pl_ and C_pl_ corresponded to the resistance and capacitance of an outer porous layer and R_bl_ and C_bl_ were the resistance and capacitance of an inner barrier (compact) layer [24,25,26,27]. From the fact that only one loop was present at the early time, it can be inferred that one compact oxide layer was formed on the TiFe alloys. With time immersed in the 30 wt% sulfuric acid electrolyte, dissolution of the oxide film occurred, as schematically shown in Figure 5. As a result, not only thinning thickness of the oxide film, but a two-layered structure of the oxide film would be formed. Such a change in the oxide film resulted in both the decreased corrosion resistance and two semicircle spectra in Figure 4b [28,29,30,31]. As discussed before, more β Ti phase coming from a higher Fe amount would impair the corrosion resistance of the TiFe alloys, and this tendency maintained even though the passive film was no longer protective after 1 day immersion in the electrolyte, which seemed to be ascribed to the α/β galvanic effect.

## 5. Conclusions

Iron played a strong role in refining grains of the pure titanium, and addition of only 0.15 wt% Fe was enough to achieve the grain size below 20 μm due to the formation of β Ti phase. Not only the microstructure, but also the added Fe exerted an influence on the corrosion properties of pure titanium. From the fact of the gradual decrease in the duration time of OCP in the passivation region, it can be inferred that the TiO_2_ stability against the highly concentrated sulfuric acid became weakened with the Fe content, leading to a decline in the corrosion resistance of pure titanium. EIS results confirmed that increasing Fe content hurt corrosion resistance, which would be ascribed to the preferential dissolution of the TiO_2_ layer around β Ti phase at grain boundaries.

## Figures and Tables

**Table 1:** Chemical compositions of the materials used in this study.

Samples	Ti	Fe	O	H	N
Ti-0.03Fe	Bal.	0.021	0.066	0.004	0.044
Ti-0.15Fe	Bal.	0.119	0.065	0.004	0.048
Ti-0.30Fe	Bal.	0.264	0.078	0.003	0.089
Ti-0.50Fe	Bal.	0.445	0.064	0.004	0.073

**Table 2:** Fitted data for parameters of equivalent circuit.

Samples		R_pl_	CPE_pl_	R_bl_	CPE_bl_
Ti-0.03Fe	0 day	1380	2.66 × 10^−3^	3.52 × 10^5^	3.12 × 10^−5^
	1 day	62.9	4.67 × 10^−4^	40.4	0.178
Ti-0.15Fe	0 day	1435	2.14 × 10^−3^	2.15 × 10^5^	3.16 × 10^−5^
	1 day	59.5	3.51 × 10^−4^	34.3	0.163
Ti-0.30Fe	0 day	1415	4.21 × 10^−4^	9.56 × 10^4^	3.21 × 10^−5^
	1 day	46.4	5.22 × 10^−4^	26.9	0.154
Ti-0.50Fe	0 day	1374	3.04 × 10^−3^	9.15 × 10^4^	3.01 × 10^−5^
	1 day	40.2	6.15 × 10^−4^	21.6	0.195

## Data Availability

The data presented in this study are available on request from the corresponding author.

## Supplementary Materials

The following are available online at https://www.mdpi.com/article/10.3390/ma14237193/s1, Figure S1: XRD diffraction patterns of Ti-Fe alloys showing presence of β-titanium phase with Fe content. Figure S2. Rietveld refinement analysis of XRD data for β Ti phase.
