# Supplementary material for: Effect of Iron Content on Corrosion Properties of Pure Titanium as Grain Refiner
Source: Materials (Basel). 2021 Nov 25;14(23):7193. doi: 10.3390/ma14237193 (PMC8658208; doi:10.3390/ma14237193)
Supplement: Supplementary file 1 [file materials-14-07193-s001.zip › materials-1474646-supplementary.pdf]

Supplementary Material

Effect of Iron Content on Corrosion Properties of Pure Titanium as Grain Refiner

Bosung Seo, Hyeon-Tae Im, Ki-Beom Park, Kwangsuk Park* and Hyung-Ki Park*

Gangwon Regional Division, Korea Institute of Industrial Technology, Gangneung-Si, Gangwon-Do, 25440, Korea; bs3863@kitech.re.kr (B.S.); iht89@kitech.re.kr (H-T.I.); hope92430@kitech.re.kr (K-B.P.)
* Correspondence: kpark63@kitech.re.kr (K.P.); mse03@kitech.re.kr (H.-K.P.)

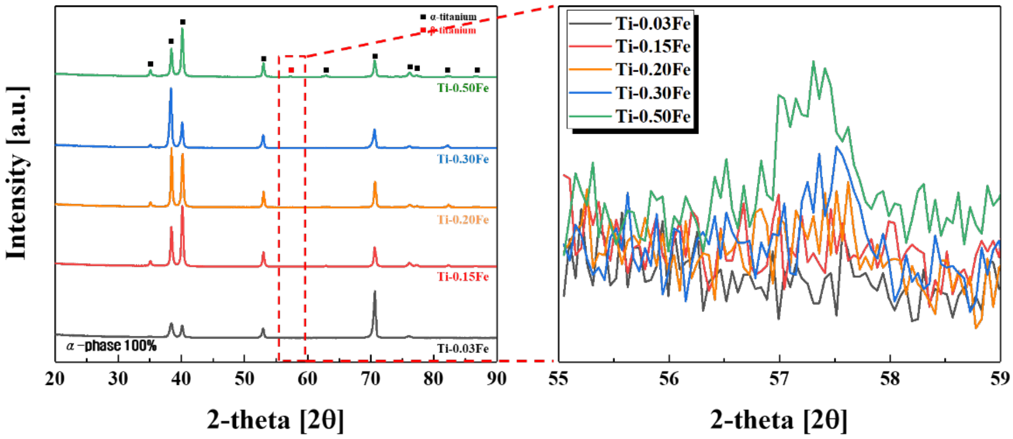

Figure S1. XRD diffraction patterns of Ti-Fe alloys, where α and β titanium phases coexisted.

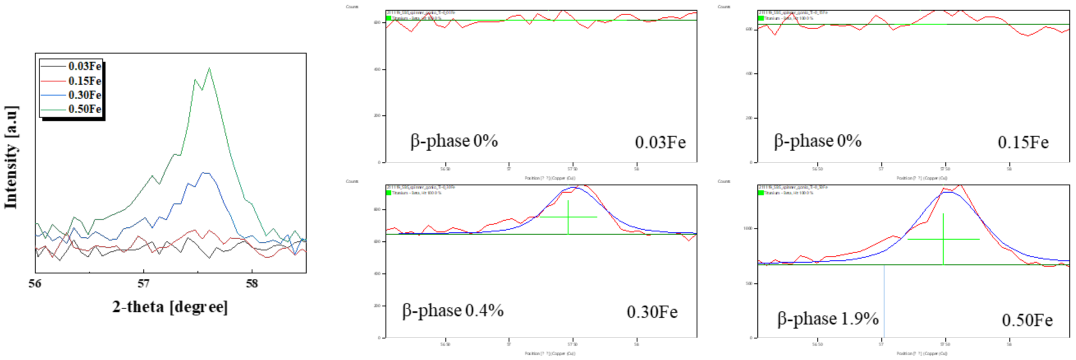

Figure S2. Rietveld refinement analysis of XRD data for β Ti phase.
